# Epigenome-wide change and variation in DNA methylation in childhood: trajectories from birth to late adolescence

**DOI:** 10.1093/hmg/ddaa280

**Published:** 2021-01-15

**Authors:** Rosa H Mulder, Alexander Neumann, Charlotte A M Cecil, Esther Walton, Lotte C Houtepen, Andrew J Simpkin, Jolien Rijlaarsdam, Bastiaan T Heijmans, Tom R Gaunt, Janine F Felix, Vincent W V Jaddoe, Marian J Bakermans-Kranenburg, Henning Tiemeier, Caroline L Relton, Marinus H van IJzendoorn, Matthew Suderman

**Affiliations:** 1 Department of Child and Adolescent Psychiatry/Psychology, Erasmus MC, University Medical Center Rotterdam, Rotterdam, The Netherlands; 2 Generation R Study Group, Erasmus MC, University Medical Center Rotterdam, Rotterdam, The Netherlands; 3 Institute of Education and Child Studies, Leiden University, Leiden, The Netherlands; 4 Lady Davis Institute for Medical Research, Jewish General Hospital, Montreal, QC, Canada; 5 Department of Epidemiology, Erasmus MC, University Medical Center Rotterdam, Rotterdam, The Netherlands; 6 Department of Psychology, Institute of Psychology, Psychiatry and Neuroscience, King's College London, London, UK; 7 MRC Integrative Epidemiology Unit, Population Health Sciences, Bristol Medical School, University of Bristol, Bristol, UK; 8 Department of Psychology, University of Bath, Bath, UK; 9 School of Mathematics, Statistics and Applied Mathematics, National University of Ireland, Galway, Ireland; 10 Molecular Epidemiology, Department of Biomedical Data Sciences, Leiden University Medical Center, Leiden, The Netherlands; 11 Department of Pediatrics, Erasmus MC, University Medical Center Rotterdam, Rotterdam, The Netherlands; 12 Clinical Child and Family Studies, Vrije Universiteit Amsterdam, Amsterdam, The Netherlands; 13 Department of Social and Behavioral Science, Harvard T.H. Chan School of Public Health, Boston, MA, USA; 14 Department of Psychology, Education and Child Studies, Erasmus University Rotterdam, Rotterdam, The Netherlands; 15 School of Clinical Medicine, University of Cambridge, Cambridge, UK

## Abstract

DNA methylation (DNAm) is known to play a pivotal role in childhood health and development, but a comprehensive characterization of genome-wide DNAm trajectories across this age period is currently lacking. We have therefore performed a series of epigenome-wide association studies in 5019 blood samples collected at multiple time-points from birth to late adolescence from 2348 participants of two large independent cohorts. DNAm profiles of autosomal CpG sites (CpGs) were generated using the Illumina Infinium HumanMethylation450 BeadChip. Change over time was widespread, observed at over one-half (53%) of CpGs. In most cases, DNAm was decreasing (36% of CpGs). Inter-individual variation in linear trajectories was similarly widespread (27% of CpGs). Evidence for non-linear change and inter-individual variation in non-linear trajectories was somewhat less common (11 and 8% of CpGs, respectively). Very little inter-individual variation in change was explained by sex differences (0.4% of CpGs) even though sex-specific DNAm was observed at 5% of CpGs. DNAm trajectories were distributed non-randomly across the genome. For example, CpGs with decreasing DNAm were enriched in gene bodies and enhancers and were annotated to genes enriched in immune-developmental functions. In contrast, CpGs with increasing DNAm were enriched in promoter regions and annotated to genes enriched in neurodevelopmental functions. These findings depict a methylome undergoing widespread and often non-linear change throughout childhood. They support a developmental role for DNA methylation that extends beyond birth into late adolescence and has implications for understanding life-long health and disease. DNAm trajectories can be visualized at http://epidelta.mrcieu.ac.uk.

## Introduction

DNA methylation (DNAm), an epigenetic process whereby DNA is modified by the addition of methyl groups, has gained increasing attention over the past few decades, due to its pivotal role in development. *In utero*, DNAm is involved in a range of essential processes, including cell differentiation (1–3), X-chromosome inactivation (4) and fetal growth (5). Its role extends well beyond birth, e.g. by maintaining cell type identity and genome stability (6–8), responding to environmental exposures (9–11), and its involvement, among many other processes, in immune (12) and neural development (13). Since it is influenced by both genetic and environmental factors (14,15), DNAm has also emerged as a key mechanism of interest for understanding the gene-environmental interplay in normal ageing and disease development.

Numerous studies have identified strong associations between DNAm and age. While most have relied on cross-sectional data (16–18), a few have utilized longitudinal measurements of DNAm within individuals (19–23). Longitudinal measurements allow one to distinguish intra-individual change from inter-individual differences in change, thereby greatly improving the power to detect change over time and to identify differences between individuals (24). Identifying and characterizing CpGs for which DNAm changes differently over time between individuals (i.e. inter-individual variation in change) is a necessary step in identifying genetic and environmental influences on the methylome as well as their potential impact on health outcomes (25). Moreover, longitudinal designs facilitate the study of non-linear trajectories (26,27), which might help to identify sensitive periods for DNAm change in development. To date, the largest epigenome-wide longitudinal study on DNAm included 385 elderly individuals who were followed up to five times over a maximum period of 18 years, identifying DNAm change at 1316 CpG (Cytosine-phosphate-Guanine) sites (19) and change of inter-individual variation at 570 CpGs (20). Yet, little is known about DNAm trajectories across early development, as existing studies in childhood DNAm typically have been limited by small sample sizes (21,23), short time-periods (22,28) or focused on specific CpGs in relation to maternal smoking (29), birthweight (30) or maternal BMI (31).

In the current study, we aim to provide a benchmark of typical epigenome-wide age-related DNAm trajectories within individuals, spanning the first two decades of life. This study combines repeated measurements of DNAm in blood at nearly half a million CpG sites across the genome from two large population-based cohorts, the Generation R Study (Generation R) and Avon Longitudinal Study of Parents and Children (ALSPAC), to form one integrated dataset with four time-points of measurement. In a series of three epigenome-wide mixed model analyses, we study linear (Model 1), non-linear (Model 2) and sex-related (Model 3) trajectories of change across development. Furthermore, we aim to identify CpGs for which trajectories vary between individuals (Models 1 and 2). Results are interpreted in the context of CpG location and biological pathways. The key findings are discussed here; full results per CpG can be freely accessed and visualized at http://epidelta.mrcieu.ac.uk/.

## Results

### Cross-cohort comparability

**Figure 1:**
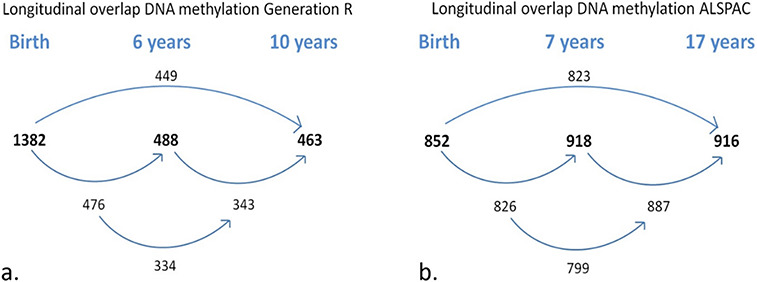
Longitudinal sample sizes for (**a**) Generation R (*N* total children = 1399, *N* total DNAm samples = 2333) and (**b**) ALSPAC (*N* total children = 949, *N* total DNAm samples = 2686). Bolded numbers represent total sample size at each time-point; non-bolded number refers to overlapping samples between time-points.

**Figure 2:**
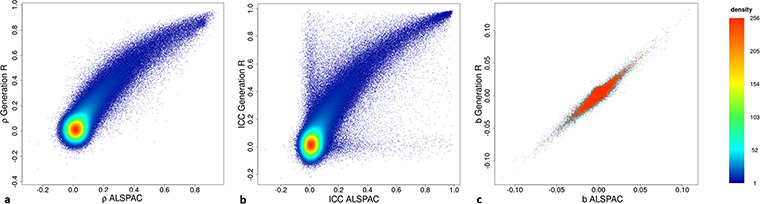
Scatterplots of within-cohort stability of DNA methylation showing (**a**) Spearman correlations, (**b**) intra-class correlation coefficients and (**c**) change estimates from birth to 6/7 years per CpG for Generation R and ALSPAC.

Sample characteristics of 1399 Generation R participants (total DNAm samples = 2333) and of 949 ALSPAC participants (total DNAm samples = 2686; Fig. 1) are provided in Supplementary Material, Table S1. After the DNAm datasets of the two cohorts underwent joint functional normalization (see Supplementary Material, Fig. S1 for distributions of mean DNAm levels), within-cohort stability of DNAm at birth and 6 or 7 years (in Generation R and ALSPAC, respectively) was compared. Stability of DNAm at individual CpG sites (437 864 autosomal sites) was estimated in three ways: relative concordance using Spearman correlations between time points, absolute concordance using intra-class correlations between time points (children with data for both time points: *n* Generation R = 476, *n* ALSPAC = 826) and change over time using change estimates from a linear mixed model (Model 1, see Materials and Methods) applied within each cohort (children with data for at least one of the two time-points: *n* Generation R = 1394, *n* ALSPAC = 944). Estimates of all stability measures for both cohorts are depicted in Fig. 2. Next, agreement of these stability estimates between the two cohorts was estimated with the Spearman (ρ) or Pearson (*r*) correlation (depending on normality of the data) across all CpGs, between the datasets. The Spearman correlation of the relative concordance was ρ = 0.62, the Pearson correlation of the absolute concordance was ρ = 0.60 and the Pearson correlation of the change estimates was *r* = 0.86, indicating strong agreement between datasets. Based on these results, the two datasets were joined to form one set with four different time-points of DNAm (birth, age 6/7, 10 and 17 years).

### Linear DNAm change from birth to early adulthood

**Figure 3:**
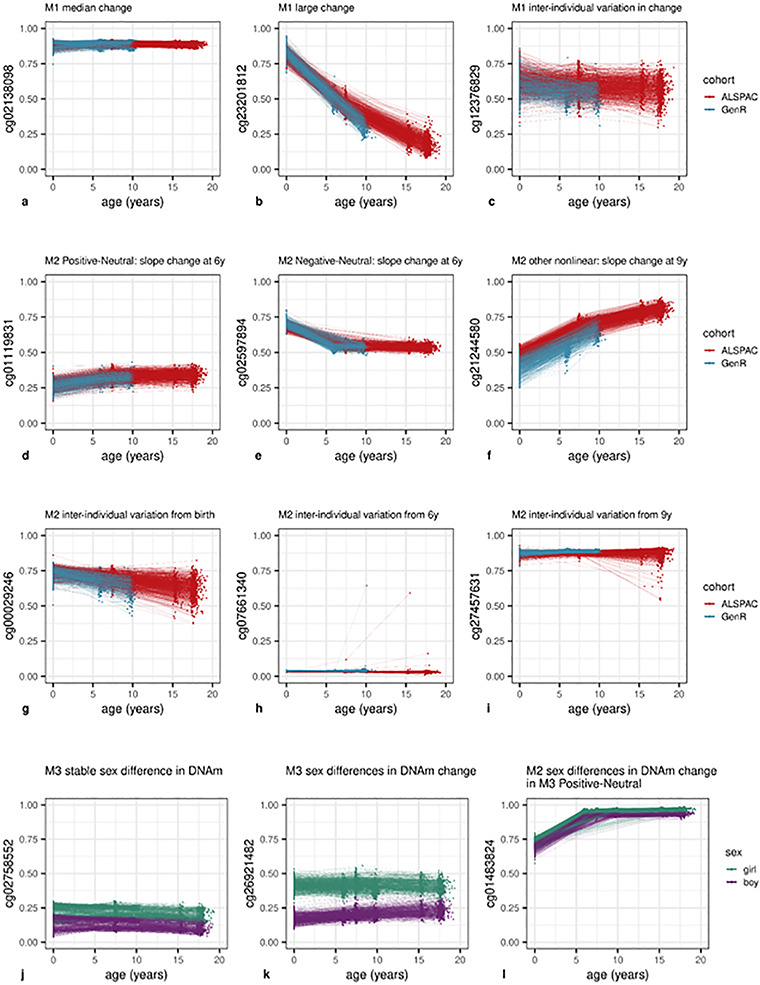
DNAm levels of selected CpG sites across childhood. Parts (**a**–**c**) show CpG sites with linear change over time (Model 1). A typical site is shown in (**a**), the site with the largest observed change in (**b**) and with inter-individual variation in DNAm change (**c**). Parts (**d**–**f**) show CpG sites with non-linear change (Model 2). A Positive-Neutral trajectory is shown in (**d**), a Negative-Neutral trajectory in (**e**) and a Positive-More Positive-Less Positive in (**f**). Parts (**g**–**i**) show CpG sites with inter-individual variation in change (Model 2). A site with slope variation from birth is shown in (**g**), slope change variation at 6 in (**h**) and slope change variation at 9 in (**i**). Parts (**j**–**l**) show CpG sites with sex-specific DNAm. A site with stable sex differences is shown in (Model 3) (**j**), sex-specific slope in (Model 3) (**k**) and sex-specific slope change at 6 in (Model 2) (**l**).

**Figure 4:**
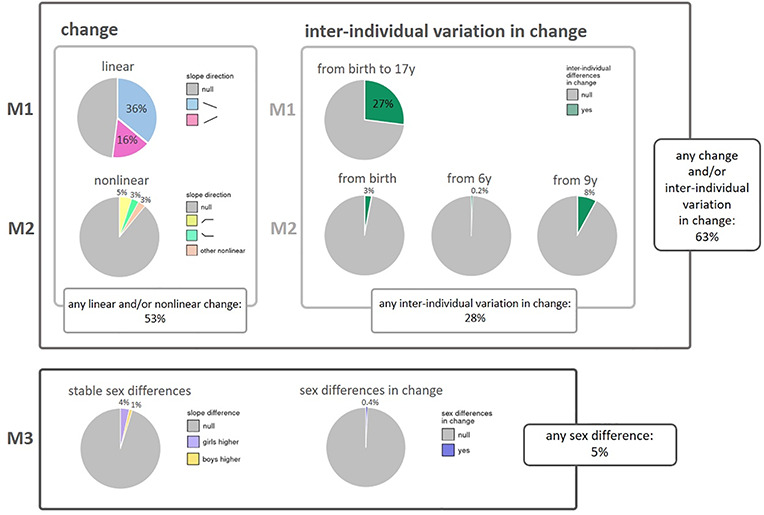
Overview of results from the three models. Model 1 (**M1**) was applied for overall change in DNA methylation and inter-individual variation in linear change; Model 2 (**M2**) for non-linear change in DNA methylation and inter-individual variation in non-linear change; and Model 3 (**M3**) for stable sex differences in DNA methylation and sex differences in change of DNA methylation (sex by time interaction). Percentages represent percentage of autosomal CpGs below Bonferroni-corrected threshold (*P* < 1 × 10^−7^).

**Figure 5:**
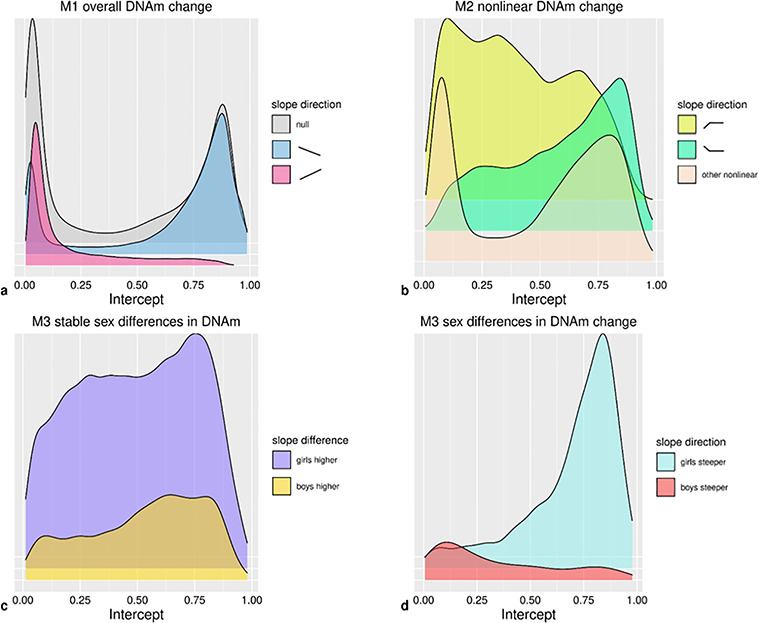
Density plots of intercepts for CpGs with (**a**) directions of change in Model 1 (*n* = 473 864); (**b**) non-linear trajectories in Model 2 (*n* = 52 043); (**c**) stable sex differences in Model 3 (*n* = 22 821); (**d**) sex differences in DNAm change in Model 3 (*n* = 1768).

Estimates of overall change in DNAm from birth to early adolescence (Model 1; see Materials and Methods) indicated linear change at 51.6% of CpGs at a Bonferroni-corrected threshold (*P* < 1 × 10^−7^) (Fig. 3a and b). Specifically, DNAm decreased over time at 35.5% of all CpGs and increased at 16.0% (Fig. 4). The mode intercept indicated that the decreasing CpGs were 88% methylated at birth (*SD* = 32%) (see Fig. 5 for the distribution of intercept for different groups of CpGs). DNAm levels for increasing CpGs typically started at 5% (*SD* = 23%).

The mode estimate of the trimodally distributed (Supplementary Material, Figure S2) DNAm change was *b* = −9.24 × 10^−4^ (with corresponding mode *SE* = 6.85 × 10^−5^), indicating an overall 0.09% DNAm decrease per year at a typical CpG site. This translates into a 1.66% decrease in DNAm over the course of 18 years. An example of a CpG site with a typical change in DNAm is depicted in Fig. 3a. The largest observed absolute change in DNAm was *b* = −3.47 × 10^−2^ (*SE* = 3.65 × 10^−4^, *P* < 9.88 × 10^−324^), indicating an overall DNAm decrease of 62.5% over 18 years (Fig. 3b). Only 22 CpGs showed an absolute change >50% over the course of 18 years (Supplementary Material, Table S2). These CpGs were mainly annotated to TSS200 regions, the gene body or intergenic regions and annotated to genes associated with global biological functions, such as protein–protein interactions [*TTC22*, (32)] or transcriptional regulation [*NFIX*, (33)] as well as more specific functions, such as synaptic scaffolding [*SHANK2*, (34)] and muscle regulation [*CSRP3*, (35)]. Associated diseases include autism spectrum disorder [*SHANK2*, (36)], cardiomyopathy [*CSRP3*, (37)] and Malan syndrome, with the latter also being characterized as an overgrowth disorder (38). With such a low number of sites that display such a large change, it follows that typically in (cord−/peripheral) blood tissue, DNAm levels for CpGs do not change from a fully unmethylated to fully methylated state, or vice versa, over the course of 18 years.

Furthermore, we observed substantial inter-individual variation in linear DNAm changes over time at 27.4% of all CpGs (i.e. random slope variance was greater than zero at Bonferroni-corrected threshold *P* < 1 × 10^−7^; Fig. 3c). On average, this variation accounted for 2.7% (*SD* = 1.5%) of all estimated inter-individual variation (for intercept, age, batch and residual; see also Supplementary Material, Table S3A) at these CpGs. At 17.3% of all CpGs, we observed both change and inter-individual variation in change.

### Nonlinear DNAm change

Model 2 (see Materials and Methods) was identical to Model 1, but permitted slope changes at ages 6 and 9 years to test for non-linear DNAm trajectories. At 11.0% of CpGs, a non-linear trajectory was detected. Specifically, at 4.8% of all CpGs, DNAm increased from birth and remained stable from 6 onward (Positive-Neutral; Fig. 3d). Second, at 3.1% of all CpGs, DNAm decreased from birth and then remained stable at 6 years (Negative-Neutral; Fig. 3e). The remaining 3.0% of all CpGs followed other non-linear trajectories (e.g. Fig. 3f), with each trajectory observed in <1.0% of all CpGs. Overall, linear and/or non-linear changes in Models 1 or 2 were observed in 52.6% of CpGs (Fig. 3), indicating that most non-linear patterns were also detected as linear patterns in Model 1.

Inter-individual differences in change (i.e. random variance in slopes) from birth onward were detected at 3.4% of all sites (Fig. 3g), inter-individual differences in slope change at 6 years in 0.2% (Fig. 3h) and inter-individual differences in slope change at 9 years at 8.2% of CpGs (Fig. 3i). For CpGs at which inter-individual differences in change from birth onwards were detected, this variation on average accounted for 3.9% (*SD* = 9.3%) of all inter-individual variation, for CpGs at which inter-individual differences in slope change at 6 years were detected, this variation on average accounted for 20.6% (*SD* = 13.2%) of all inter-individual variation and for CpGs at which differences in slope change at 9 years were detected, this variation on average accounted for 23.3% (*SD* = 17.6%) of all inter-individual variation (for a complete overview of inter-individual variation within all CpGs, see Supplementary Material, Table S3B). We note that CpGs with inter-individual differences in change at 6 years often contained a few extreme outliers. These outliers, however, were found across cohorts and batches and were often found within individuals across time-points, indicating that these outliers seem to reflect true DNAm values rather than technical issues. Inter-individual differences in slope (change) at each time-point were detected more often at CpGs with an increasing rather than decreasing overall DNAm change in Model 1 (*P* ≤ 2.37 × 10^−144^). At last, both Positive-Neutral and Negative-Neutral changes coincided more often with inter-individual variation from birth (*P* < 9.88 × 10^−324^). Any inter-individual differences in change, detected by Models 1 or 2, were observed at 27.9% of CpGs. In total, Models 1 and 2 detected age-related change whether linear, non-linear or inter-individual differences in change at 62.8% of all CpG sites (Fig. 3).

### Sex differences in longitudinal DNAm and DNAm change

According to Model 3 (see Materials and Methods), sex differences in DNAm were present at 4.9% of (autosomal) CpGs (Fig. 3). Specifically, stable longitudinal sex differences (main sex effects) were observed at 4.8% of all (autosomal) CpGs (Fig. 3j), and sex differences in DNAm change (sex by age interaction effects) were found at 0.4% of all (autosomal) CpGs (Fig. 3k). At sites with stable sex differences, DNAm levels were higher in girls at 3.6% (Fig. 3j) and lower at 1.2% of CpG sites. DNAm at sites with higher DNAm in girls tended to increase over time, whereas DNAm at sites with higher DNAm in boys tended to decrease (*P* = 4.20 × 10^−205^). Most commonly (at 0.2% of all CpGs), DNAm was higher in girls at birth but DNAm in boys increased at a higher rate.

Both CpGs with stable sex differences and those with sex differences in DNAm change were less likely to show inter-individual variation in DNAm change than other sites (20.8 versus 27.5% and 18.1 versus 27.3%; *P* = 5.36 × 10^−111^ and *P* = 7.57 × 10^−18^). Finally, CpGs with stable sex differences or sex differences in DNAm change detected in Model 3 were much more likely to follow an overall Positive-Neutral trajectory of DNAm change detected in Model 2 than other CpG sites were (24.2% of CpGs with stable sex differences followed a Positive-Neutral trajectory versus 3.8% of other CpGs and 53.9% of CpGs with sex differences in DNAm change followed a Positive-Neutral trajectory versus 4.6% of other CpGs; *P* < 9.88 × 10^−324^, *P* < 9.88 × 10^−324^; Fig. 3l). Albeit less prominently so, CpGs with stable sex differences or sex differences in DNAm change also more often followed a Negative-Neutral trajectory than other CpGs did (stable sex differences: 5.0 versus 3.0%, *P* = 5.43 × 10^−62^; sex differences in DNAm change: 7.7 versus 3.1%, *P* < 7.11 × 10^−28^).

### Follow-up analyses

**Figure 6:**
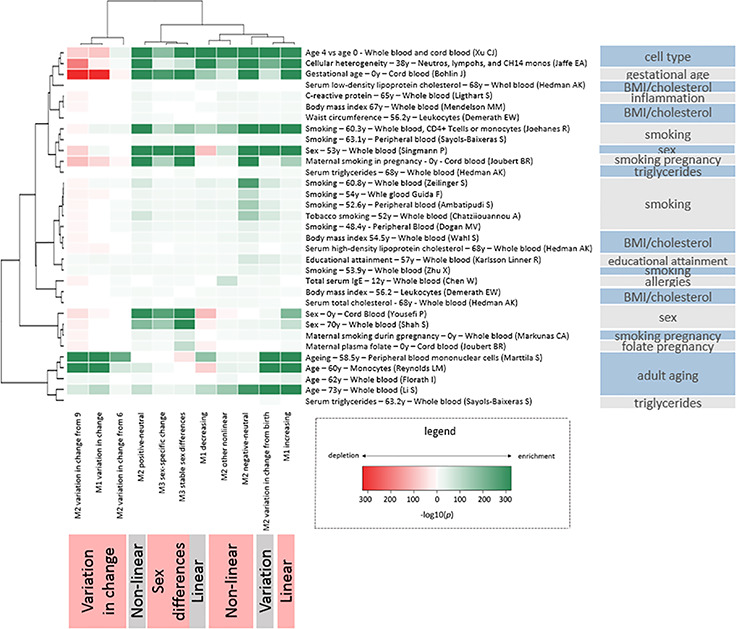
Enrichment of age-related trajectories in EWASs.

Follow-up analyses were performed to understand how different types of age-related DNAm trajectories are distributed across the genome (Supplementary Material, Tables S4–S6) and in association with genetic variance (Supplementary Material, Tables S7–S9). Additionally, to understand how different types of DNAm change are represented in epigenetic age estimators, we studied their enrichment in Horvath’s (39) and Hannum’s (40) age estimators (Supplementary Material, Tables S10 and S11). All reported enrichments have significance below a Bonferroni-corrected threshold of *P* < 3.29 × 10^−4^, corrected for the number of chi-square tests (*n =* 152). We further report the enrichment of Gene Ontology (GO) pathways (nominal *P* < 0.05) for genes annotated to CpG sites in each trajectory (Supplementary Material, Tables S12–S14). At last, we study the enrichment of age-related DNAm trajectories in reported hits of different epigenome-wide association studies (EWASs) (Fig. 6). All reported EWAS enrichments are below a Bonferroni-corrected threshold of *P* < 1.38 × 10^−04^, corrected for the number of Fishers’ exact tests (*n* = 363; Supplementary Material, Table S15).

#### Patterns of DNAm change and CpG location

CpG sites with DNAm change associated patterns were labelled by gene associated regions, CpG island associated regions as well as enhancer elements. Although many exceptions exist, low levels of DNAm in the promoter area but high levels of DNAm in the gene body are generally associated with increased gene transcription (41,42). CpGs annotated to TSS200 regions more often showed an overall DNAm increase (Model 1) than other CpGs (19.0 versus 15.6%), whereas CpGs annotated to the gene body more often showed an overall DNAm decrease than other sites (38.8 versus 33.7%). TSS200 CpGs showed less inter-individual variation in overall DNAm change than other sites (22.2 versus 28.1%), whereas gene body CpGs showed somewhat more inter-individual variation in overall DNAm change than other sites (28.9 versus 26.5%).

Promoter areas often coincide with CpG islands (43). Here, 63.3% of TSS200 CpGs were also annotated to CpG islands. As in TSS200 areas, CpGs annotated to CpG islands had lower DNAm levels [mode M1 intercept 2.4% (*SD* = 30.2%)], and more often showed an overall DNAm increase than other sites (25.2 versus 12.0%). DNAm sex differences were especially present in the shores of CpG islands compared with all other island associated regions (stable sex differences: 7.5 versus 4.0%, sex differences in DNAm change: 0.6 versus 0.3%).

Enhancers act on promoters to regulate gene transcription (44). CpGs annotated to enhancer elements (2.0% of CpGs) tended to have low DNAm levels (mode M1 intercept 5.07%; *SD* = 31.4%) and then increased with age more than other CpGs (23.9 versus 15.9%). Inter-individual variation in change from birth was more common at enhancer sites than at other sites (5.6 versus 3.3%).

#### Genetic associations with patterns of DNAm change

We looked up methylation quantitative trait loci *in cis* (*cis* meQTLs) and *in trans* (*trans* meQTLs) (45) and CpGs located on a single nucleotide polymorphism (polymorphic CpGs) (46) to investigate possible relationships between genetic variation and DNAm trajectories and sex differences. CpGs with *cis* (30.0% of CpGs) and *trans* (2.1% of CpGs) meQTLs were more likely to have increasing DNAm than other sites (*cis*: 23.4 versus 12.9% at other sites; *trans*: 27.5 versus 15.8%) and to exhibit more inter-individual variation in overall DNAm change (*cis*: 30.8 versus 25.9%; *trans*: 29.4 versus 27.3%). This inter-individual variation tended to appear from birth onwards (*cis*: 5.4 versus 2.5%; *trans*: 13.3 versus 3.1%) and not from 9 years onwards (*cis*: 7.7 versus 8.4%; *trans*: 4.7 versus 8.2%). CpG sites with meQTLs were more likely to exhibit stable DNAm sex differences (*cis*: 9.0 versus 3.0%; *trans*: 15.2 versus 4.6%) and sex differences in DNAm change than other sites (*cis*: 0.7 versus 0.2%; *trans*: 2.0 versus 0.3%).

Polymorphic CpGs (or for Infinium I, single nucleotide polymorphisms at site of single base extension) (14.0% of all CpGs) more often had decreasing (42.72 versus 34.35%) rather than increasing DNAm (9.16 versus 17.15%) than other CpGs had. Furthermore, there was less inter-individual variation in the slope change at 9 years (5.86 versus 8.54%) and less stable sex differences were found at polymorphic CpGs (3.61 versus 5.01%).

In sum, CpGs linked to meQTLs more often had increasing DNAm and DNAm sex differences, whereas polymorphic CpGs less often had increasing DNAm and DNAm sex differences. Both CpGs linked to meQTLs and polymorphic CpGs less often showed inter-individual variation in the slope change at 9 years.

#### Epigenetic age estimators

‘Epigenetic age acceleration’ is a term coined to indicate the deviation of chronological age from age as estimated by an ‘epigenetic clock’ and is associated with disease risk and mortality (47). In our own sample (time-points 6/7 to 17 years), the median absolute difference (MAD) between epigenetic age and chronological age for two often-used epigenetic clocks, those of Horvath (39) (*n* = 353 CpGs) and Hannum *et al.* (40) (*n* = 71), was rather large but similar to most other published studies (39,40,48,49), with an MAD of 2.9 and 3.8 years, respectively. A previous study found epigenetic age to be non-linearly related to chronological age in both childhood and adulthood (50). However, to use epigenetic age acceleration as an unbiased predictor, one would expect a linear association between the epigenetic clock and age, and thus for the clock to contain few CpGs that change nonlinearly over time. Additionally, to detect age acceleration, one would expect that change in DNAm at these CpGs would also vary between individuals. Here, we tested the enrichment of our DNAm trajectories in the epigenetic clocks of Horvath (*n* = 353 CpGs) and Hannum *et al.* (*n* = 71).

CpGs in both epigenetic clocks more often showed non-linear DNAm patterns than other sites did (26.4% in Horvath’s clock showed non-linear patterns; 45.1% in Hannum’s clock did). Slope changes in DNAm were found both at the age of 6 (21% in Horvath; 40.8% in Hannum) and 9 years (7.1% in Horvath, 8.4% in Hannum). No enrichment for inter-individual variation in DNAm change was found within Horvath’s clock, whereas CpGs in Hannum’s clock showed enrichment for inter-individual variation in DNAm change from birth compared with other sites (16.9 versus 3.4%) and from 9 years onwards (28.2 versus 8.2%). Based on this, we conclude neither that both epigenetic clocks did meet our expectations on the specific inclusion of linearly changing probes, nor that CpGs in Horvath’s epigenetic clock are enriched for inter-individual variation in DNAm change.

#### Functional associations

Enrichment of GO categories was tested for genes linked to CpGs with different DNAm trajectories. In short, genes annotated to CpGs with overall decreasing DNAm levels were enriched in immune-developmental functions, whereas those annotated to CpGs with increasing levels were enriched in neurodevelopmental functions. This pattern seemed even more pronounced at genes annotated to non-linear Negative-Neutral and Positive-Neutral CpGs, with the former more often associated to immune-development and the latter to neurodevelopment. Genes linked to CpGs with stable sex differences and sex differences in DNAm change were enriched in pathways associated with sexual development, such as genital development, as well as pathways associated with neurodevelopment. Genes linked to CpGs with sex difference in DNAm change were also enriched in functions related to tooth and hair development.

#### Enrichment in EWASs

We further investigated the functional relevance of CpG sites with age-related DNAm trajectories by testing enrichment with published EWAS associations (Fig. 6) (28,51–76). Unsupervised clustering of the enrichments shows that CpG sites with inter-individual variation in change over time have distinct enrichments and cluster differently from those with age-associated change that is consistent among individuals. The CpG sites of each age-associated DNAm trajectory were enriched with published age associations in adulthood. Multiple smoking EWAS clustered together with enrichment patterns exhibiting strongest enrichments among CpG sites with negative-neutral trajectories and mostly weak enrichments among CpG sites with inter-individual variation in change. Furthermore, despite adjusting for cell count heterogeneity in our models, we observed enrichments of CpG sites that differ by white blood cell (WBC) type among sites following nearly all age-associated trajectories; however, the increases in proportions were small. Finally, we observed enrichments of CpG sites associated with gestational age and prenatal smoking with sex-specific DNAm.

## Discussion

In this study, we described changes in DNAm levels through the first two decades of human life. We examined DNAm levels per CpG by their linear association with age, their non-linear trajectories and inter-individual variation in change, as well as sex differences and CpG characteristics.

We found that about half of sites change: consistent linear and/or non-linear DNAm change was found at 53% of sites. We further found that over a quarter of sites, 28% were characterized by substantial inter-individual differences in the direction of this change. DNAm sex differences were present, but not abundant: 5% of autosomal sites displayed different DNAm levels or differences in change over time for girls and boys.

Specifically, we determined that DNAm at 52% of the measured methylome have some form of linear change from birth to late adolescence, with DNAm decreasing at 36% and increasing at 16% of CpGs. CpGs with decreasing DNAm tended to have high levels of DNAm and were more often located in gene bodies. CpGs with increasing levels of DNAm tended to have low levels of DNAm and were more likely to be located in promoter regions and at enhancers. The predominance of decreasing CpGs is in agreement with literature on epigenome-wide DNAm and age in cross-sectional research on children and adults (18,77), as well as with longitudinal research in adults (19).

Non-linear DNAm trajectories were detected at 11% of CpGs, mostly involving changes in DNAm from birth to age 6 years, after which DNAm was more stable. We note that this could be due to cord blood being used to generate DNAm profiles at birth, whereas peripheral blood was used at later ages. A previous study (23) including eight children showed that the cord blood DNAm profile at birth clustered separately from later peripheral profiles, after which DNAm changed gradually from 1 to 2.5–5 years. Such differences between DNAm in cord and peripheral blood might be due to uncaptured differences in WBC composition, as well as to different gene-regulatory functioning in the intra-uterine versus extra-uterine environment.

Sites with decreasing levels of DNAm, both with or without slope changes around the age of 6 years, were functionally enriched for immune-developmental pathways, and sites with increasing levels of DNAm, both with or without slope changes, were enriched for neurodevelopmental pathways. Since these observations were based on blood DNAm, it remains to be studied what roles genes linked to neurodevelopmental pathways play in in blood, or to what extent DNAm trajectories in blood mirror those in neural tissue.

To our knowledge, we are the first to study inter-individual differences in DNAm change. Inter-individual differences in linear DNAm trajectories were found at 27% of CpGs, indicating change at different rates or directions for different individuals. Such sites tended to have overall increasing rather than decreasing levels of DNAm from birth to 18 years. More research would be needed to understand why these to qualities so often co-occur in CpGs. Inter-individual differences in non-linear DNAm trajectories were most often found in the slope change at 9 years (8% of CpG sites), indicating that most inter-individual differences in DNAm emerge after the first decade of life. More research is needed to understand if the direction of change in this period is determined by stimuli during that period, or rather by preceding, perhaps cumulative, exposures. However, it is clear that, given the high proportion of CpG sites with inter-individual variation in DNAm change over time that we have observed, it is important to restrict the range of ages of children included a single EWAS. Specific limits should be discussed given the rapidly growing number of studies generating DNAm profiles across childhood (78).

Stable sex differences were found at 5% of autosomal CpGs, and sex differences in DNAm change were found at 0.4% of all CpGs. In general, if there were stable sex differences, girls had higher levels of DNAm (4% of all CpGs); in case of sex differences in DNAm change, boys had an accelerated upward change (0.2% of all CpGs). The direction of stable sex differences detected is congruent with a cross-sectional study on newborns, in which girls had higher DNAm levels than boys for the large majority of the 3031 significant autosomal CpGs (69). Sex-discordant associations with age seemed to be more prevalent from birth to age 6 years than afterwards, suggesting that any phenotypic sex differences associated to DNAm would be established in early childhood. Sex-associated CpGs showed less inter-individual variation in change and tended to be associated with meQTLs, and therefore, more likely to be genetically influenced. Their enrichment in the shores of CpG islands, areas at which DNAm has been associated with tissue differentiation and tissue-specific gene expression (79), is consistent with the critical role that these processes play in sexual differentiation. Studies into sex differences in epigenetic regulation might want to focus on these locations.

We also found the other DNAm trajectories to be arranged throughout the genome in a non-random fashion. Earlier studies (41,80) have shown that, for active genes, lower DNAm towards the promoter area (TSS200) and higher DNAm in the gene body relate to increased gene transcription. Here, we add the observation that promoter DNAm tends to increase and gene body DNAm tends to decrease with age. From this finding, one might infer that a downregulation of gene expression takes place from birth to late adolescence. Enrichment analyses of published EWAS associations further showed that different traits and exposures exhibited distinct enrichment patterns among DNAm trajectories. For example, there were clear differences between smoking and BMI-related traits. Enrichment of sites with DNAm sex differences in EWASs on prenatal maternal smoking is consistent with studies finding that prenatal smoking affects traits, such as birth weight (81), brain development (82,83) and attention (84) differently in boys and girls. Clustering for prenatal maternal smoking EWASs also showed enrichment for CpGs with consistent change among individuals, not for CpGs with inter-individual variation in change. This may suggest a link with the well-known effects of prenatal smoking on childhood development since consistent DNAm change is more likely related to development or aging programming than inter-individual variation. This may explain why changes associated to prenatal smoking persist throughout life (85). Notably, this pattern of change without inter-individual variation is visible in cg05575921, the *AHRR* CpG site strongly and persistently associated with prenatal smoking (86,87) (Supplementary Material, Fig. S3; http://epidelta.mrcieu.ac.uk).

As a *post hoc* sensitivity analysis, we recomputed our main results, excluding a list of cross-reactive probes (46), and compared these to our main results. In most cases, percentages of CpGs representing the various DNAm trajectories were significantly different when excluding the cross-reactive probes, but absolute percentages did not change more than 0.5%. The trajectory that had a somewhat larger relative change in percentage was the CpGs in which stable sex differences were detected (4.8% in all CpGs; 4.5% excluding cross-reactive probes), which is in line with earlier reports that autosomal cross-reactive probes may reflect false DNAm differences between the sexes (46,88). However, since we found that this group of CpGs is enriched for associated genes involved in sexual development, we conclude that the large majority of the CpGs identified to have sex differences are not affected by cross-reactivity.

We found that DNAm trajectories for CpGs included in epigenetic clocks employed to estimate age or age acceleration did not meet our expectations. For example, we observe that over one-quarter and nearly one-half of the CpG sites included in the Horvath and Hannum clocks, respectively, follow non-linear DNAm trajectories in childhood. Given the widespread use of clocks to investigate biological aging, further investigation is warranted to better understand how, and perhaps if, associations using these clocks should be interpreted in child DNAm profiles.

We note three main limitations of our findings. First, the use of different tissue types (cord blood and peripheral blood) could account for some of the differences between birth and later time points, e.g. sites that increased or decreased between birth and 6, but did not show change after that. Although enrichment analyses further showed that these sites were enriched with sites differentially methylated between WBC types, the enrichments represent small proportions of sites with linear or non-linear change identified in our study. Generation of DNAm profiles of a single tissue type or cell type collected across childhood, or the measurement of WBC proportions instead of estimation of proportions, would be needed to disentangle this issue further. Second, since DNAm at 9 years was measured only in Generation R and at 17 years only in ALSPAC, DNAm differences from 9 to 17 may be to some extent driven by batch effects or cohort differences. This may explain some of the inter-individual differences in slope changes at 9 towards 17 years. However, the high level of agreement in both stability and change among the corresponding time points of the two cohorts is reassuring. Moreover, it is not entirely surprising that inter-individual variation in directionality of change was higher for the largest age interval. This interval, furthermore, encompasses the period of adolescent development, a time in which many inter-individual phenotypic differences arise. Finally, it should be noted that the current study only included children of European ancestry. Considerable DNAm differences have been found between populations (89–91), but research on age-associated DNAm differences is scarce. One study (92) reported evidence for overlap in age-associated CpGs in two African populations with studies on European-ancestry populations, but more research is needed to map the generalizability of longitudinal DNAm changes among different populations.

## Conclusions

In the first comprehensive CpG-by-CpG characterization of DNAm from birth to late adolescence, we found that DNAm at more than half of the studied CpG sites changes consistently between individuals and that considerable inter-individual variation in change exists. Furthermore, characteristics, such as child sex, CpG location, genetic variants, and environmental and disease traits, have distinct associations with patterns of DNAm change. Further analysis of these patterns is made readily available at http://epidelta.mrcieu.ac.uk/, which we hope can be used in future studies to test developmental hypotheses that promote our understanding of the developmental nature of DNAm, its role in gene functioning and the associated biological pathways leading to health and disease.

## Materials and Methods

### Setting

Data were obtained from two population-based prospective birth cohorts: the Dutch Generation R Study (Generation R) and the British Avon Longitudinal Study of Parents and Children (ALSPAC). Pregnant women residing in the study area of Rotterdam, The Netherlands, with an expected delivery date between April 2002 and January 2006 were invited to enrol in Generation R. A more extensive description of the study can be found elsewhere (93). The Generation R Study is conducted in accordance with the World Medical Association Declaration of Helsinki and has been approved by the Medical Ethics Committee of the Erasmus Medical Center, Rotterdam. Informed consent was obtained for all participants.

Pregnant women residing in the study area of former county Avon, UK, with an expected delivery date between April 1991 and December 1992 were invited to enrol in the ALSPAC study. Detailed information on the study design can be found elsewhere (3,94). The ALSPAC website contains the details of all available data through a fully searchable data dictionary and variable search tool (http://www.bristol.ac.uk/alspac/researchers/our-data/). Ethical approval for the study was obtained from the ALSPAC Ethics and Law Committee and the Local Research Ethics Committees. Consent for biological samples has been collected in accordance with the Human Tissue Act (2004). Informed consent for the use of data collected via questionnaires and clinics was obtained from participants following the recommendations of the ALSPAC Ethics and Law Committee at the time.

### Study population

In the Generation R Study, 9778 pregnant mothers had 9749 live-born children. For a subsample of 1414 children, DNAm data were collected at birth and/or 6 years and/or 10 years of age. This subsample consisted of participants with parents born in the Netherlands [European ancestry (95) confirmed for all children with genetic data available (95.4%)]. Fifteen sibling pairs were present in the dataset. From each pair, one sibling with the lowest number of DNAm measurements, or otherwise randomly, was excluded, resulting in a sample with 1399 children (with 2333 DNAm samples; see below).

In the ALSPAC study, 15 247 pregnant mothers gave birth to 14 973 live-born children. DNAm at birth and/or 7 years and/or 17 years was available for a subsample of 1003 children as part of the Accessible Resource for Integrated Epigenomic Studies (ARIES) study (96). From this sample, 48 children with non-European ancestry as based on genetic principle component analysis and 6 children with missing data on gestational age were excluded, resulting in a sample of 949 children with DNAm data (with 2686 DNAm samples; see below).

### DNA methylation

Cord blood was drawn after birth for both cohorts, and peripheral blood was drawn at a mean age of 6.0 (*SD* = 0.5) and 9.8 (*SD* = 0.3) years for Generation R, and 7.5 (*SD* = 0.2) and 17.1 (*SD* = 1.0) years for ALSPAC. Both cohorts made use of the EZ-96 DNAm kit (shallow) (Zymo Research Corporation, Irvine, CA) to perform bisulfite conversion on the extracted leukocytic DNA. Samples were further processed with the Illumina Infinium HumanMethylation450 BeadChip (Illumina Inc., San Diego, CA) to analyze DNAm.

In Generation R, quality control was performed on all 2467 available DNAm samples with the CPACOR workflow (97). Arrays with observed technical problems, such as failed bisulfite conversion, hybridization or extension as well as arrays with a mismatch between sex of the proband and sex determined by the chromosome X and Y probe intensities, were removed from subsequent analyses. Additionally, only arrays with a call rate >95% per sample were processed further, resulting in 2355 samples, 22 of which belonged to half of an excluded sibling pair; hence, 2333 samples were carried forward into normalization.

In ALSPAC, quality control was performed on 6057 samples (3286 belonging to children, 2771 to their mothers), using the *meffil* package (98) in R version 3.4.3 (99). After removing samples with mismatched genotypes, mismatched sex, incorrect relatedness, low concordance with samples collected at other time points, extreme dye bias and poor probe detection, 5337 samples remained, 2845 of which belonging to children, used in the current study.

Although we removed low-quality samples in part based on high proportions of probes with undetectable signal or low bead numbers, we did not afterward remove any probes due to poor signal. If we apply a typical probe-exclusion procedure, with detection *P*-value >0.01 or low bead count for more than 10% of the samples, we would remove only 1414 or 0.3% of all autosomal probes. The removal of such a small number of probes would not change our results in any noteworthy way.

To minimize cohort effects as much as possible, we normalized both cohorts together as a single dataset. Functional normalization (10 control probe principal components, slide included as a random effect) was performed with the *meffil* package in R (98). Normalization took place on the combined Generation R and ALSPAC set comprising a total of 5178 samples for a total of 485 512 CpGs. One-hundred and fifty-nine ALSPAC samples belonging to non-European children or children with missing data on gestational age were excluded, leading to a final ALSPAC set of 2686 samples (for 949 children). Together with 2333 samples for Generation R (of 1399 children), they formed a combined set of 5019 samples (of 2348 children.)

Analyses were restricted to 473 864 autosomal CpGs. DNAm levels were operationalized as beta values (β values), representing the ratio of methylated signal relative to the sum of methylated and unmethylated signal measured per CpG. In a *post hoc* sensitivity analysis, recomputed our main results excluding a list of cross-reactive probes [*n* = 28 298; 5.9% of all included CpGs (46)] and compared them to our main results (Supplementary Table S16).

### Covariates

Sample plate number (*N* = 29 in Generation R and *N* = 31 in ALSPAC) was used to correct for batch effects, which was added as a random variable in the model (see below). WBC composition was estimated with the reference-based Bakulski method (100) for cord blood and Houseman method (101) for peripheral blood (Supplementary Material, Table S17). Nucleated red blood cells were not further analyzed due to its specificity to cord blood, leaving CD4+ T-lymphocytes, CD8+ T-lymphocytes, natural killer cells, B-lymphocytes, monocytes and granulocytes. Other covariates included gestational age in weeks, sex of the child, and cohort.

### Statistical analyses

*Step 1: Assessing cross-cohort comparability in DNA methylation stability.*

To ascertain comparability among the two cohorts, we compared within-cohort DNAm stability between the time points that were present in both cohorts—i.e. birth and 6/7 years (Generation R/ALSPAC, respectively).

Longitudinal stability per CpG within each cohort was assessed by studying estimates of concordance and change. For concordance, DNAm data were first residualized within each cohort for all variables present in the longitudinal models except the ‘cohort’ variable, in order to remove between-cohort differences due to other covariates. Concordance was then measured both with Spearman correlation (data at most CpGs is not normally distributed) as a measure of relative concordance and with intra-class correlations as a measure of absolute concordance (children with data for both time points: *n* Generation R = 476, *n* ALSPAC = 826). Longitudinal change from birth to 6/7 years was assessed by studying the estimates of the change in DNAm per year by applying Model 1 (see below) within each cohort (children with data for at least one of the two time-points: *n* Generation R = 1394, *n* ALSPAC = 944).

In a second step, cross-cohort comparability was assessed with Spearman (ρ) correlation of concordance estimates of the CpGs of each cohort (which were not normally distributed) and Pearson correlations (*r*) among the change estimates of the CpGs of each cohort (which were normally distributed).

*Step 2: Longitudinal modelling of DNA methylation using combined Generation R and ALSPAC data.*

The combined Generation R and ALSPAC dataset had four time points of collection (birth, age 6/7, 10 and 17 years). We fit three linear mixed models to CpG site DNAm across the genome to identify (i) linear change over time (Model 1); (ii) non-linear change over time (Model 2) and (iii) sex differences in change over time (Model 3). Both fixed and random effects were examined to allow for inter-individual variation in DNAm patterns over time. The models are described in detail below.

*Model 1: Linear change.* This model was applied to identify CpGs that show an overall change in DNAm from birth to 18 years (i.e. fixed age effect), as well as CpGs with inter-individual differences in change during that time (i.e. random age effect). The Model 1 is defined as follows:

M1: *M_ijk_ = β*_0_ + *u_0i_* + *β*_1_Age*_ij_* + *u*_1*i*_Age*_ij_* + *u*_0*k*_ + covariates + *ϵ_ijk_*

    *ϵ_ijk_ ~ N*(0,*σ_ϵ_^2^*)

    *u*_0*i*_*~ N*(0,*σ_0i_^2^*)

    *u*_1*i*_*~ N*(0,*σ_1i_^2^*)

    *u*_0*k*_*~ N*(0,*σ_0k_^2^*)

(i.e.: CpG ~ (1|Child ID) + Age + (Age|Child ID) + (1|Sample Plate) + covariates).

Here, participants are denoted by *i*, time points by *j* and sample plates by *k*. *M* denotes DNAm level, *β_0_* fixed intercept, *u_0i_* random intercept, *β_1_* fixed age coefficient, *u_1i_* random age coefficient and *u_0k_* random intercept for sample plate. Hence, *β_1_* represents the average change in DNAm per one year. Variability in this change among individuals was captured with *u_1i_.* To avoid problems with model identification, the random slope of age was uncorrelated to the random intercept (i.e. a diagonal random effects matrix was used).

*Model 2: Non-linear change.* To identify non-linear changes in DNAm, we extended Model 1 to allow slope changes at ages 6 and 9 (30,31)

M2: *M_ijk_ = β*_0_ + *u*_0*i*_ + *β*_1_Age*_ij_* + *β*_2_(Age*_ij_−*6)^+^ + *β*_3_(Age*_ij_−*9)^+^ +*u*_1*i*_ Age*_ij_* + *u*_2*i*_ (Age*_ij_−*6)^+^ + *u*_3*i*_ (Age*_ij_−*9)^+^ + *u*_0*k*_ + covariates + *ϵ_ijk_*

    *ϵ_ijk_ ~ N*(0,*σ_ϵ_^2^*)

    *u*_0*i*_*~ N*(0,*σ_0i_^2^*)

    *u*_1*i*_*~ N*(0,*σ_1i_^2^*)

    *u*_2*i*_*~ N*(0,*σ_2i_^2^*)

    *u*_3*i*_*~ N*(0,*σ_3i_^2^*)

    *u*_0*k*_*~ N*(0,*σ_0k_^2^*)

(i.e. CpG ~ (1|Child ID) + Age + Age-6 + Age-9 + (Age|Child ID) + (Age-6|Child ID) + (Age-9|Child ID) + (1|Sample Plate) + covariates),

Where *a*^+^ = *a* if *a* > 0 and 0 otherwise, so that *β_2_* represents the average change in DNAm per year from 6 years of age onward, after accounting for the change per year from birth onward, as denoted by *β_1_.* Likewise, *β_3_* represents the average change in DNAm per year from 9 years of age onward, after accounting for the change per year from 6 years of age onward. Hence, with those variables, we are able to detect slope changes at 6 and 9 years old. These slope changes were used to identify different types of non-linear patterns. With *u*_2*i*_ and *u*_3*i*,_ the inter-individual variation in slope changes at 6 and 9 years were captured, respectively. General linear hypothesis testing (102) was applied to our fitted models to determine if there were changes in DNAm per year from 6 to 9 years and from 9 to 18 years.

*Model 3: Sex differences in change:* To identify CpGs for which DNAm changes differently over time for boys and girls, we applied the following model:

M3: *M_ijk_ = β*_0_ + *u*_0*i*_ + *β*_1_Age*_ij_* +*u*_1*i*_ Age*_ij_* + *β*_2_ Sex*_i_* Age*_ij_* + *u*_0*k*_ + covariates + *ϵ_ijk_*

    *ϵ_ijk_ ~ N*(0,*σ_ϵ_^2^*)

    *u_0i_ ~ N*(0,*σ_u_^2^*)

    *u_1i_ ~ N*(0,*σ_1i_^2^*)

    *u_0k_ ~ N*(0,*σ_0k_^2^*)

(i.e. CpG ~ (1|Child ID) + Age + (Age|Child ID) + Age^*^Sex + (1|Sample Plate) + covariates),

Here, Sex*_i_* denotes the sex of child *i*. Both main and interaction effects for sex were studied.

The three mixed models were fitted using maximum likelihood estimation in *R* with the *lme4* package (103). Continuous covariates (WBCs, gestational age) were z-score standardized. Random slopes were kept uncorrelated with random intercepts and the NLopt optimizer was used, enabling us to improve computational speed compared with the default settings. *P*-values for the fixed effects were computed with a z-test. *P*-values for random slopes of the Age effects were obtained by refitting the model without the random slope and comparing the fit estimates of the two models with a likelihood ratio test. Within each model, *P*-value thresholds were Bonferroni-corrected for the number of tested CpGs (i.e. to *P* < 1 × 10^−7^).

*Step 3: Functional characterization of CpGs with comparable patterns of change.*

To interpret the functionality of the age-related DNAm patterns from the three models, CpG sites adhering to 8 different age-related patterns (M1 linear change and inter-individual variation in linear change, M2 non-linear trajectories and inter-individual variation in change from birth in slope change at 6 and 9 years, and M3 stable sex differences and sex differences in DNAm change) were tested for enrichment in the following:

(i) gene-relative genomic regions (TSS1500, TSS200, 5’UTR, 1st exon, gene body, 3’UTR and intergenic regions) as indicated by the UCSC Genome Browser (104) in the Illumina HumanMethylation450 v1.2 Manifest (Illumina Inc.),
(ii) CpG island-relative genomic regions (N shelf, N shore, CpG island, S shore, S shelf and open sea regions) as indicated by the UCSC Genome Browser (104) in the Illumina HumanMethylation450 v1.2 Manifest (Illumina Inc.),
(iii) enhancer elements as those expressed in whole blood, peripheral blood mononuclear cells, natural killer cells, CD4+ T cells, CD8+ T cells, monocytes, neutrophils, eosinophils or B cells (105),
(iv) *cis* and *trans* meQTLs identified by the BIOS consortium (45) and polymorphic CpGs (46) and
(v) inclusion in two well-known epigenetic age estimators (39,40).

Altogether, these encompassed 19 enrichment analyses for eight variables. Enrichment was tested using χ^2^-tests of unequal proportions. The enrichment *P*-value threshold was Bonferroni-corrected for multiple tests (i.e. *P* < 3.29 × 10^−4^ for 8 × 19 = 152 tests). Second, we tested the enrichment of GO categories for genes linked to CpG sites surviving adjustment for multiple tests (*P* < 1 × 10^−7^) for each of the main variables of interest. Gene annotation (closest genes, <1500 base pairs from transcription start site) was based on the Illumina HumanMethylation450 v1.2 Manifest (Illumina Inc.). The analysis was adjusted for gene size and pruned for near-identical terms [see elsewhere for a full description (106)]. For completeness, terms with nominal *P* < 0.05 were reported. At last, we tested the enrichment of age-related DNAm trajectories (11 different age-related patterns: M1 decreasing, increasing and inter-individual variation in linear change, M2 Positive-Neutral, Negative-Neutral, other non-linear, inter-individual variation in change from birth, in slope change at 6 and 9 years and M3 stable sex differences and sex differences in DNAm change) in EWASs on age, prenatal smoking, smoking, cardiovascular-associated traits, C-reactive protein, allergies, educational attainment and cellular heterogeneity. EWAS summary statistics were retrieved from the EWAS Catalog (http://www.ewascatalog.org/) and studies were included when performed with the 450 K array in peripheral or cord blood, resulting in 33 EWASs. Enrichment was tested with Fisher’s exact tests; the enrichment *P*-value threshold was Bonferroni-corrected for multiple tests (i.e. *P* < 1.38 × 10^−4^ for 11 × 33 = 363 tests).

## Supplementary Material

Mulder_201203_EpiDelta_BigChangers_SupplementaryTable_2_ddaa280

Mulder_201203_EpiDelta_EWASenrichments_SupplementaryTable_15_ddaa280

Mulder_201203_EpiDelta_GO_BP_SupplementaryTable_12_ddaa280

Mulder_201203_EpiDelta_GO_CC_SupplementaryTable_13_ddaa280

Mulder_201203_EpiDelta_GO_MF_SupplementaryTable_14_ddaa280

Mulder_201203_EpiDelta_SupplementaryMaterial_ddaa280
